# Sodium Glucose Cotransporter-2 Inhibitors in Non-Diabetic Kidney Disease: Evidence in Experimental Models

**DOI:** 10.3390/ph17030362

**Published:** 2024-03-11

**Authors:** Giovanna Castoldi, Raffaella Carletti, Francesca Barzaghi, Michela Meani, Giovanni Zatti, Gianluca Perseghin, Cira R. T. Di Gioia, Gianpaolo Zerbini

**Affiliations:** 1 Dipartimento di Medicina e Chirurgia, Università degli Studi di Milano-Bicocca, 20900 Monza, Italy; francesca.barzaghi20@gmail.com (F.B.); m.meani2@campus.unimib.it (M.M.); giovanni.zatti@unimib.it (G.Z.); gianluca.perseghin@unimib.it (G.P.); 2 AOU Policlinico Umberto I, 00161 Roma, Italy; raffaella.carletti@uniroma1.it; 3 Clinica Ortopedica, Fondazione IRCCS San Gerardo dei Tintori, 20900 Monza, Italy; 4 Dipartimento di Medicina Interna e Riabilitazione, Policlinico di Monza, 20900 Monza, Italy; 5 Dipartimento di Scienze Radiologiche, Oncologiche e Anatomopatologiche, Istituto di Anatomia Patologica, Sapienza Università di Roma, 00161 Roma, Italy; cira.digioia@uniroma1.it; 6 Unita’ Complicanze del Diabete, Diabetes Research Institute, IRCCS Istituto Scientifico San Raffaele, 20132 Milano, Italy; zerbini.gianpaolo@hsr.it

**Keywords:** SGLT2 inhibitors, non-diabetic kidney disease, experimental models of non-diabetic nephropathies

## Abstract

Sodium glucose cotransporter 2 (SGLT2) inhibitors are a class of glucose-lowering agents widely used for the treatment of type 2 diabetes mellitus. A number of clinical trials in type 2 diabetic patients with different degrees of renal impairment have clearly demonstrated that SGLT2 inhibitors reduce the progression rate of diabetic kidney disease. Furthermore, recent studies have shown that SGLT2 inhibitors also exert a protective effect in the case of non-diabetic kidney disease. Consequently, it has been hypothesized that the nephroprotective activity of these drugs could exceed the canonical impact on glycemic control and that the resulting beneficial effects could be the consequence of their pleiotropic properties (proven reduction of inflammation, fibrosis, oxidative stress and sympathetic nervous activity) both at systemic and tissue levels, suggesting that the efficacy of these drugs could also be extended to non-diabetic nephropathies. This review focuses on the nephroprotective effects of SGLT2 inhibitors in different experimental models of non-diabetic kidney disease. The different glucose-independent mechanisms potentially implemented by SGLT2 inhibitors to ultimately protect the non-diabetic kidney are described in detail, and conflicting results, when present, are discussed.

## 1. Introduction

**Figure 1:**
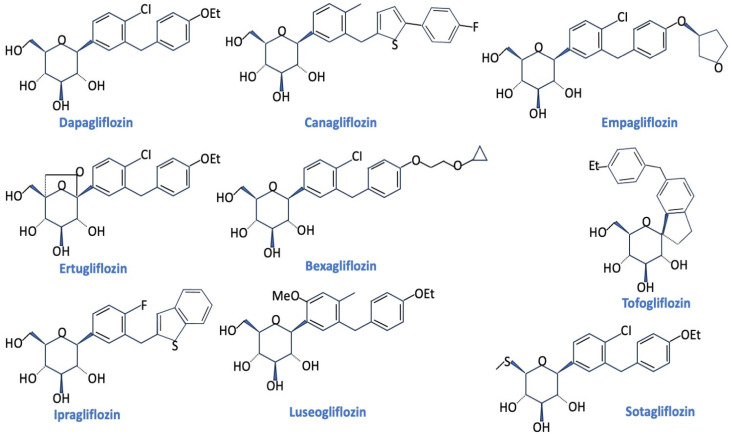
Chemical structures of principal FDA-approved SGLT2 inhibitors (Dapagliflozin, Canagliflozin, Empagliflozin, Ertugliflozin) and others that have been recently developed [4].

Sodium glucose co-transporter 2 (SGLT2) inhibitors are widely used as blood glucose-lowering drugs for the management of type 2 diabetes mellitus (Figure 1) [1,2,3,4,5].

**Figure 2:**
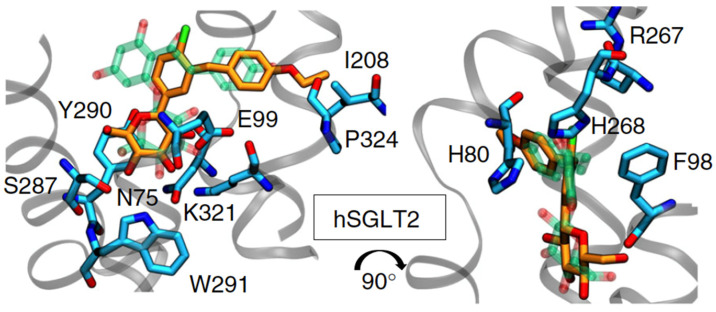
Predicted binding mode of Dapagliflozin (**left**) to SGLT2 (**right**), as published in [5].

The way in which SGLT2 inhibitors bind to SGLT2 has been identified, and an example (binding of dapagliflozin to SGLT2) is shown in Figure 2.

**Figure 3:**
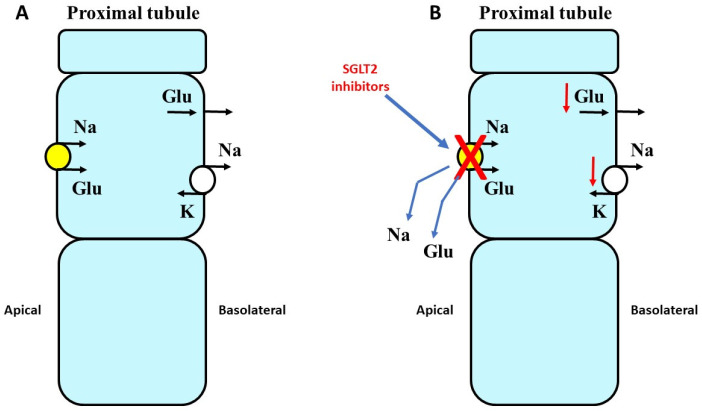
Schematic representation of sodium/glucose cotransporter 2 (SGLT2) action. (**A**) SGLT2 is localized in the proximal tubule where it is in charge of the reabsorption of sodium and glucose. (**B**) In the case of the blockade of SGLT2 by specific inhibitors, increasing amounts of sodium and glucose are lost with urine.

When administered to individuals affected by diabetes, these drugs inhibit the renal reabsorption of glucose by blocking the sodium-coupled glucose transporter 2 (SGLT2) selectively expressed in the proximal tubule (Figure 3).

This mechanism gives rise to glycosuria, a phenomenon that results in a lower blood glucose concentration and, as a consequence, in an improvement of the metabolic control [6].

Besides decreasing plasma glucose levels, SGLT2 inhibitors also interfere with multiple hemodynamic and non-hemodynamic activities, leading to additional effects, including weight loss, blood pressure lowering, reduction in uric acid concentration and attenuation of glomerular hyperfiltration, promoted by osmotic diuresis and glycosuria-coupled natriuresis [6,7,8,9].

At the initial stages of diabetic nephropathy, in particular in the case of hyperglycemia, an increased amount of glucose reaches the proximal tubule, and it is reabsorbed, along with sodium, through the SGLT2. As a result, the amount of sodium that, inside the nephron, may move ahead to finally reach the macula densa is significantly reduced. This lower concentration of sodium triggers the tubuloglomerular feedback, a mechanism that, with the final aim to increase the osmolarity of urine, induces the constriction of the efferent and the dilation of the afferent arteriole, thus resulting in a higher intraglomerular pressure and an increased glomerular filtration rate. Chronic hyperfiltration determines, in the long run, a progressive damage of glomerular barrier and of its permselective properties toward proteins, causing proteinuria and, as a consequence, the development of chronic kidney disease [10,11,12].

The inhibition of SGLT2 blocks the reabsorption of glucose and sodium by the proximal tubule, and, consequently, it increases the concentration of glucose and sodium reaching the macula densa. This results in the inhibition of the tubulo-glomerular feedback, remodeling of renal arteriolar constriction and, finally, normalization of the glomerular filtration rate [13]. The reduction of the glomerular filtration rate will decrease the albumin excretion rate and, as a consequence, will also slow down the rate of progression of the diabetic kidney disease.

Clinical studies have demonstrated that SGLT2 inhibitors maintain nephroprotective effects even when a glomerular filtration rate is significantly reduced [14,15], thus suggesting that, in addition to glycemic control, other mechanisms, such as reduction of inflammation, oxidative stress and sympathetic nervous activity, might be implicated in organ protection induced by SGLT2 inhibitors [13,16,17,18].

These well-established pleiotropic effects of SGLT2 inhibitors strongly support the hypothesis that these drugs could also have a clinical efficacy in the case of renal diseases not related to diabetes.

The aim of this review is to summarize the experimental evidence that supports or fails to support the nephroprotective efficacy of SGLT2 inhibitors in non-diabetic kidney diseases, and to describe at least some of the possible mechanisms involved in SGLT2 inhibitors-driven nephroprotection in the absence of hyperglycemia.

## 2. SGLT2 Inhibitors in Experimental Models of Non-Diabetic Kidney Disease

The renal beneficial actions of SGLT2 inhibitors in diabetic rats and mice have been extensively reviewed [19].

To demonstrate the renal protective effects of SGLT2 inhibitors in the absence of hyperglycemia and, therefore, their beneficial effects beyond blood glucose reduction, the effects of SGLT2 inhibitors on renal morphology and function have been investigated in experimental models of non-diabetic renal disease.

### 2.1. Hypertensive Nephropathy

In angiotensin II (Ang II)-dependent hypertension, caused by Ang II administration in rats (200 ng/kg/min, sub cutis, for two weeks), treatment with the SGLT2 inhibitor empagliflozin (10 mg/kg/day) promoted natriuresis throughout the entire experimental period but did not significantly modify the Ang II-dependent blood pressure increase, glomerular filtration rate, kidney/body weight ratio and urinary oxidative stress marker 8-isoprostane. Empagliflozin administration in Ang II-treated rats prevented the onset of renal glomerular and tubulo-interstitial fibrosis, the expression of type I and type IV collagen and the increase in inflammatory cell infiltration (Table 1) [20].

Ang II administration at a higher dose (400 ng/kg/min, sub cutis, for two weeks) caused kidney damage and an increase in SGLT2 cotransporter expression [21]. Interestingly, in these experimental conditions, the inhibition of SGLT2 in the presence of AT1 receptor blockade showed a major effect in preventing kidney damage as compared to the SGLT2 inhibitor empagliflozin (10 mg/kg/day) alone or AT1 blocker alone (Table 1) [21]. In fawn-hooded hypertensive rats and in Goldblatt (2 kidney-1 clip) hypertensive rats, empagliflozin administration (10 mg/kg/day for eight weeks) did not reduce creatinine, markers of inflammation and oxidative stress, and it did not prevent proteinuria, but it actually increased it (Table 1) [22].

### 2.2. Salt-Sensitive Hypertension and Uninephrectomy DOCA Salt Models

In uninephrectomized rats with non-diabetic salt-sensitive hypertension, the SGLT2 inhibitor empagliflozin (20 mg/kg/day for 3 weeks) reduced blood pressure and renal inflammatory infiltrates and restored the impaired pressure natriuresis phenomenon (Table 1) [23]. Differentially, always in uninephrectomized rats with non-diabetic salt-sensitive hypertension, empagliflozin (10 mg/kg/day for eight weeks) did not provide nephroprotection, not reducing proteinuria, oxidative stress and inflammation (Table 1) [22]. In female Dahl salt-sensitive rats, dapagliflozin (2 mg/kg/day for 3 weeks) prevented the progression of salt-induced hypertension, as observed in male salt-sensitive rats. SGLT2 inhibition did not modify creatinine clearance but reduced kidney fibrosis (Table 2) [32].

In Dahl salt-sensitive rats, dapagliflozin (0.1 mg/kg/day for 6 weeks) decreased albuminuria, renal fibrosis, inflammation and oxidative stress, stimulating the protective pathway of the renin–angiotensin system by the increase in the AT2 and Mas receptor expression (Table 2) [33]. Of interest, in uninephrectomy DOCA salt mouse models with different genetic predispositions to develop glomerular damage, empagliflozin treatment (30 mg/kg/day) did not show nephroprotective effects both in terms of urinary albumin excretion and damage to the renal parenchyma (Table 1) [24].

### 2.3. Polycystic Kidney Disease

In PCK rats, an orthologous model of autosomal recessive polycystic kidney disease (PKD), the administration of the SGLT2 inhibitor dapagliflozin (10 mg/kg/day) for 6 weeks caused glycosuria, transient hyperfiltration and an increase in diuresis, albuminuria, kidney weight and cyst volume (Table 2) [34]. Conversely, dapagliflozin administration (10 mg/kg/day) for 5 weeks to Han: SPRD rats with PKD did not modify cyst growth, renal macrophages infiltration and interstitial fibrosis, but it increased urinary glucose excretion and reduced albuminuria (Table 2) [35]. In this PKD model, the combined SGLT1/2 inhibitor phlorizin (400 mg/kg/day for 5 weeks) improved renal function, as assessed by creatinine clearance measurements, induced natriuresis, reduced urinary albumin excretion rate and lowered kidney weight and cyst volume (Table 3) [44].

### 2.4. Obesity and Insulin Resistance

Insulin resistance represents a risk factor for chronic kidney disease, promoting kidney damage through several mechanisms, such as the increase in the advanced glycation end-products (AGEs) and chronic inflammation. Insulin resistance was induced in rats by adding 10% fructose in drinking water for 20 weeks. Rats with insulin resistance showed hyperglycemia and renal fibrosis characterized by a renal downregulation of sirtuin-1 (Sirt 1) expression and by an increase in tumor necrosis factor-α (TNF-α) and transforming growth factor-β1 (TGFβ-1). The SGLT2 inhibitor empagliflozin (30 mg/kg/day for 4 weeks) and tumor necrosis factor-a antibody infliximab (IFX) upregulated Sirt 1 expression and reduced renal fibrosis. Interestingly, combination treatment (empagliflozin plus infliximab) was more effective than monotherapy in reducing fibrosis (Table 1) [25].

High-fat diet (HFD)-fed rats had both obesity and impaired renal function, thus giving rise to a prediabetic rat model. SGLT2 inhibitors lowered body weight, creatinine clearance and microalbuminuria. Furthermore, the SGLT2 inhibitor dapaglifozin (1 mg/kg/day for 4 weeks) suppressed renal inflammation and fibrosis, subsequently inducing a significant decrease in obesity-related renal injury. The SGLT2 inhibitor treatment attenuated renal inflammation, as indicated by a lower expression of NF-κB, p65, TNF-α, IL-1β, COX-2 and iNOS, and renal tubulointerstitial fibrosis. Dapaglifozin improved renal oxidative stress by lowering malonaldehyde (MDA) and increasing glutathione (GSH) content. In HFD rats, renal endoplasmic reticulum (ER) stress, shown by the upregulation of GRP78, CHOP, Calpain 2 and Caspase-12 expression, was reduced by dapagliflozin treatment. SGLT2 inhibition prevented apoptosis by restoring anti-apoptosis protein Bcl-2 and downregulating pro-apoptotic protein Bax, cytochrome c, cleaved caspase-3 and the number of TUNEL-positive cells (Table 2) [36].

In high-fat/high-fructose diet (HFF)-induced insulin resistant rats, characterized by lipid accumulation in the kidney and liver, the SGLT2 inhibitor dapagliflozin (1 mg/kg/day for 4 weeks), alone or in combination with atorvastatin, improved HFF-induced insulin resistance and renal oxidative stress, inflammation and fibrosis related to lipotoxicity (Table 2) [37].

### 2.5. Cisplatin-Induced Nephropathy

Cisplatin (CP) is a chemotherapy drug used to treat different types of cancers with nephrotoxic side effects. The SGLT2 inhibitor canagliflozin reduced CP-induced increase in plasma creatinine, urea, uric acid and urinary albumin/creatinine ratio. Treatment with SGLT2 inhibitors decreased the CP-induced increase in plasma levels of inflammatory cytokines TNF-α, IL-6 and IL-1β, of clusterin and cystatin C. Furthermore, in the case of CP-induced nephrotoxicity, canaglifozin (10–30 mg/kg/day for 10 days) reduced urinary 8-OHdG, 8-isoprostane and L-FABP concentrations and increased the antioxidant activity of glutathione reductase, total antioxidant capacity, catalase and superoxide dismutase. Histologic sections evidenced that the administration of SGLT2 inhibitors blunted tubulointerstitial and glomerular damages in CP-induced nephrotoxicity (Table 4) [49].

In vitro, canagliflozin reduced CP-induced renal proximal tubular cell apoptosis enhancing the activation of AKT. In vivo, canagliflozin (20 mg/kg/day for 1 week before cisplatin injection and for 1 day after cisplatin injection) reduced cisplatin uptake by kidney tissue, apoptosis and tubular injury (Table 4) [50].

### 2.6. Gentamicin-Induced Nephropathy

Gentamicin (GNT) is an antibiotic used to treat gram-negative bacterial infections. Its adverse effects include kidney damage through the formation of reactive oxygen species (ROS) and the subsequent activation of inflammatory pathways resulting in acute kidney injury. The SGLT2 inhibitor dapaglifozin (10 mg/kg/day for 2 weeks) reduced the increase in the serum concentrations of urea and creatinine and the worsening of proteinuria induced by gentamicin in rat. Furthermore, the administration of dapaglifozin attenuated the GNT-induced increase in oxidative stress by decreasing MDA and increasing GSH levels in renal tissue. The effect of the SGLT2 inhibitor dapaglifozin on apoptosis-associated miRNAs evidenced a significant elevation in miR-21 expression and a reduction in miR-181a expression in GNT-treated rats. The administration of SGLT2 inhibitors also controlled the apoptosis signaling pathway Bax/Bcl-2 by suppressing the immunohistochemical reaction of cytochrome c and caspase-3. The histological sections showed improvement in renal tissue morphology by restoring the renal glomerular and tubular structures (Table 2) [38].

### 2.7. Adenine-Induced Nephropathy

Kidney disease can be induced in rodent models by administering an adenine-rich diet. Treatment with the SGLT2 inhibitor canaglifozin (10 and 25 mg/kg/day for 35 days) reduced the adenine-induced elevation of plasma concentrations of creatinine, urea and uric acid. Urinary albumin and the albumin–creatinine ratio were increased in the adenine-fed rats and were mitigated after the administration of SGLT2 inhibitors. Creatinine clearance was reduced in adenine-treated rats and was not modified by canagliflozin treatment. Canaglifozin treatment increased renal antioxidants, such as superoxide dismutase, catalase, glutathione reductase and total antioxidant capacity, which were decreased by adenine administration. Furthermore, SGLT2 inhibitors had an anti-inflammatory effect through the reduced expression of the plasma levels of inflammatory markers, including interleukin 6, interleukin 1 beta, tumor necrosis factor alpha, clusterin and cystatin C, in adenine-fed rats. Finally, SGLT2 inhibitors improved all histological changes observed in adenine-fed rats, including renal tubular alterations and inflammatory cells infiltration and fibrosis of the interstitium (Table 4) [51].

The administration of the SGLT2 inhibitor ipragliflozin at a low dose (0.03 and 0.1 mg/kg/day) for 4 weeks in adenine-induced chronic kidney disease in mice did not affect crystal deposits in tubules but reduced renal dysfunction, tubular dilatation and fibrosis (Table 3) [45]. In adenine-induced chronic kidney disease uninephrectomized rats fed a high-salt diet, the SGLT2 inhibitor luseogliflozin (10 mg/kg/day) did not modify the high levels of blood urea nitrogen and tubular injury but reduced hypertension and attenuated the sympathetic nerve activity (Table 3) [46].

### 2.8. Protein-Overload Nephropathy

Protein-overload induced by bovine serum albumin (BSA) causes loss of glomerular barrier’s permselective properties, leading to proteinuria and glomerular disease. In mice with BSA protein-overload proteinuria, treatment with dapagliflozin (1.5 mg/kg/day for 3 weeks) induced natriuresis and lowered blood pressure and proteinuria but did not affect the glomerular filtration rate. In dapaglifozin-treated mice, the decrease in urinary protein excretion was associated with the amelioration of glomerular lesions and a reduction in podocyte dysfunction and glomerular and interstitial macrophage infiltration. BSA injection increased the expression of SGLT2 in podocytes, suggesting a potential mechanism of action of SGLT2 inhibitors. In proteinuric glomerular disease in mice with BSA protein-overload proteinuria, dapagliflozin promoted glomerular protection to the same degree as the ACE inhibitor lisinopril (Table 2) [39].

### 2.9. Oxalate-Induced Nephrocalcinosis 

C57BL/6N mice fed with an oxalate-rich diet developed nephrocalcinosis-related tubular atrophy and interstitial fibrosis, preserving the glomerular structure. The administration of the SGLT2 inhibitor empagliflozin (10 mg/kg/day for 1 or 2 weeks) did not affect the progressive decline of renal function, indicated by the increase in plasma creatinine and urea and by the decrease in the glomerular filtration rate. Empaglifozin had no influence on the kidney/body weight ratio in oxalate-treated mice. This drug did not show beneficial effects on renal tubular injury and inflammation evaluated by measuring kidney injury molecule-1, tissue inhibitor of metalloproteinase 2, insulin-like growth factor binding protein 7, tumor necrosis factor-α and NLRP3 expression. Empaglifozin did not counteract the development of interstitial fibrosis and the expression of fibroblast-specific protein-1, transforming growth factor-β2, collagen 1α1 and fibronectin-1 (Table 1) [26].

### 2.10. Cyclosporin Nephropathy

In Sprague Dawley rats, under a low-salt diet, Cyclosporine-A (CsA, 15 mg/kg/day, intraperitoneal injection) was administered for 4 weeks to induce nephropathy. The rats treated with cyclosporine-A showed an increase in blood pressure, which was reduced by the administration of the SGLT2 inhibitor empagliflozin (10 mg/kg/day for 4 weeks). As compared to the control rats, CsA administration caused an increase in glomerular and tubulo-interstitial fibrosis, renal inflammatory infiltrates and tyrosine hydroxylase expression, used as a marker of sympathetic activation. All these parameters were reduced by empagliflozin administration, suggesting nephroprotective effects of SGLT2 inhibition in CsA nephropathy (Table 1) [27].

### 2.11. Adriamycin-Induced Kidney-Injury

Adriamycin-induced nephropathy is characterized by proteinuria, progressive glomerulosclerosis, interstitial fibrosis and inflammation. In balb/c mice, adriamycin administration caused proteinuria and kidney injury, which were not modified by SGLT2 inhibition. In fact, dapagliflozin administration (1 or 3 mg/kg/day for 2 weeks) did not ameliorate proteinuria, glomerulosclerosis, tubulointerstitial inflammation, fibrosis, tubular casts and tubular atrophy, suggesting that SGLT2 inhibition has no nephroprotective effects in adriamycin-induced kidney injury (Table 2) [40].

### 2.12. Renal Ischemia-Reperfusion Injury

Renal ischemia-reperfusion (IR)-induced injury causes acute kidney injury (AKI) and increases the risk of developing renal interstitial fibrosis. Treatment with SGLT2 inhibitors ameliorates renal functional parameters by decreasing serum creatinine and urea levels. SGLT2 inhibitor dapaglifozin (10 mg/kg/day for 2 days) attenuates renal tubulo-interstitial damages and promotes anti-apoptotic action, as indicated by the higher renal Bcl-2 expression and reduced Bax expression and TUNEL-positive cells. SGLT2 inhibition increased hypoxia-inducible factor 1 (HIF1) expression, a key protein that regulates cellular adaptation to hypoxia, which is also markedly increased in renal IR injury (Table 2) [41].

In renal IR-injury mice, the administration of the SGLT2 inhibitor luseogliflozin (30 mg/kg/day for 1 week) reduced renal hypoxia, endothelial rarefaction and interstitial fibrosis, evidenced by attenuated collagen-specific Sirius Red staining and TGF-β expression. After I/R injury, the SGLT2 inhibitor luseogliflozin reduced renal capillary rarefaction and the detachment of pericytes from endothelial cells. After controlateral uninephrectomy at 4 weeks after I/R, luseogliflozin administration increased creatinine clearance. Finally, after I/R injury, the administration of SGLT2 inhibitors enhanced renal vascular endothelial growth factor-A expression (Table 3) [47].

### 2.13. Unilateral Ureteric Obstruction

Unilateral ureteric obstruction (UUO) is a classic experimental model of renal interstitial fibrosis. In UUO rats, the SGLT2 inhibitor empagliflozin (10 mg/kg/day; prophylactic treatment: starts 1 week before surgery and continues for 2 weeks after operation; immediate treatment: starts immediately after UUO and continues for 2 weeks; delayed treatment: starts 1 week after UUO and continues for 2 weeks) significantly lowered blood urea and creatinine, whereas it had no effect on body weight. UUO in rats caused the onset of tubular damage, inflammation and interstitial fibrosis through the activation of inflammatory (NF-κB, TLR4) and fibrotic signaling pathways (TGF-β1, α-SMA, Wnt, CTGF and fibronectin). UUO downregulated klotho expression, a transmembrane protein with renal anti-fibrotic action. Empaglifozin administration reduced the levels of NF-κB, TLR4, α-SMA, TGF-β1, Wnt-1 and fibronectin, preventing the decline of renal function, UUO-induced fibrosis and tubular injuries, and increased renal klotho activity. Prophylactic and immediate treatments with empagliflozin have a greater nephroprotective action compared to delayed treatment (Table 1) [28].

### 2.14. Subtotal 5/6 Nephrectomy

Subtotal nephrectomized Sprague Dawley rats were treated with the SGLT2 inhibitor TA-1887 (10 mg/kg/day for 10 weeks) to investigate the activity of the systemic and intrarenal renin–angiotensin system (RAS). The administration of TA-1887 did not significantly lower body weight but augmented kidney/body weight ratio. TA-1887 did not affect functional and hemodynamic parameters, including plasma urea or creatinine levels, creatinine clearance, urinary albumin excretion and blood pressure. Furthermore, TA-1887 did not change the renal tissue injury, as indicated by glomerular PAS- or tubulointerstitial Azan-positive areas. The SGLT2 inhibitor did not modify plasma renin activity, plasma angiotensinogen level and angiotensin II content in the kidney in 5/6 nephrectomized rats (Table 3) [48].

The 5/6 nephrectomized rats developed proteinuria, hypertension and a decline in glomerular filtration rate, which was not modified by treatment with the SGLT2 inhibitor dapaglifozin (0.5 mg/kg/bi-daily for 12 weeks). Treatment with dapagliflozin did not reduce glomerulosclerosis, tubulointerstitial fibrosis and the overexpression of TGFβ 1, a profibrotic factor, in the kidney of 5/6 nephrectomized rats, suggesting that SGLT2 inhibition did not promote nephroprotection in this model of progressive non-diabetic kidney disease (Table 2) [42].

In 5/6 nephrectomized rats under a high-salt diet, the cardio-renal effects of the SGLT2 inhibitor empagliflozin (0.6 mg/kg/bi-daily for 8 weeks) were compared to the AT1 blocker telmisartan and placebo. A low-dose empagliflozin treatment reduced renal fibrosis in 5/6 nephrectomized rats as compared to the placebo group. The antifibrotic effect of empagliflozin was similar to that obtained with telmisartan. Interestingly, in 5/6 nephrectomized rats under a high-salt diet, the antifibrotic effects of the SGLT2 inhibitor and AT1 blocker seemed to be independent of the tubuloglomerular feedback (TGF), as measured by urinary adenosine excretion, a surrogate parameter for TGF activation (Table 1) [29]

The SGLT2 inhibitor dapaglifozin (1 mg/kg/day for 8 weeks) did not modify renal hemodynamic function, the decline in GFR, and it did not have any anti-proteinuric effects on 5/6 nephrectomized rats. The SGLT2 inhibitor dapaglifozin lowered blood pressure but did not change glomerulosclerosis and tubulointerstitial macrophage infiltration (Table 2) [43].

In 5/6 nephrectomized rats, empagliflozin administration (15 mg/kg/day for 6 weeks) reduced blood creatinine and urinary albumin excretion as compared to placebo. Single-cell transcriptome analysis of kidneys showed that beneficial effect on kidney function and the renal antifibrotic effects of empagliflozin were mediated by the inhibition of M2 macrophage polarization and by the reduction of inflammatory signals (Table 1) [30].

### 2.15. Isoprenaline Sympathetic Nervous System Stimulation

The effects of the SGLT2 inhibitor canagliflozin were investigated in a rat model of chronic sympathetic nervous system hyperstimulation obtained by the administration of the non-selective β-AR agonist isoprenaline, which causes renal injury by the stimulation of oxidative stress and inflammation. Canagliflozin administration (5 mg/kg/day for 1 week) improved kidney function in isoprenaline-treated rats by reducing oxidative stress, inflammation, fibrosis and apoptosis (Table 4) [52].

### 2.16. Cardiorenal Syndrome

In cardiorenal syndrome, caused by chronic kidney disease and dilated cardiomyopathy, induced by 5/6 nephrectomy and doxorubicin treatment, respectively, the SGLT2 inhibitor empagliflozin (20 mg/kg/day) preserved heart function, reduced myocardial fibrosis, lowered plasma creatinine and urinary protein/creatinine ratio and improved renal tubular structure (Table 1) [31].

## 3. Discussion

SGLT2 inhibitors entered the market a few years ago, being considered as a novel antidiabetic drug that reduces blood glucose levels by increasing urinary glucose excretion (glycosuria). This novel pharmacologic approach was very effective, and SGLT2 inhibitors, also because of their very limited side effects, rapidly became among the most used drugs in the anti-diabetic field.

With time, it became clear that the beneficial effects of SGLT2 inhibitors exceed their glucose-lowering properties. The reason is still poorly understood; SGLT2, as a functional transporter, is present in reasonable amounts only in the proximal tubule, and, in the absence of hyperglycemia, most of the renal and extra-renal effects (see above for details) of this class of drugs remain difficult to explain.

We also have to take into account the evidence that SGLT2 inhibitors, when administered to different experimental models of non-diabetic nephropathy, may give rise to different, sometimes conflicting, results, as summarized in Table 1, Table 2, Table 3 and Table 4. In fact, in some cases, SGLT2 inhibitors show beneficial effects by protecting the kidneys and improving functional parameters (plasma creatinine and urea, glomerular filtration rate, urinary protein excretion) and tissue parameters (kidney weight, glomerular and tubular structural dysfunctions, interstitial fibrosis, inflammatory cellular infiltration and oxidative stress) [20,21,23,25,27,28,29,30,31,32,33,35,36,37,38,39,41,44,45,47,49,50,51,52]. In other cases, however, SGLT2 inhibitors seem to have no protective impact on the kidney [22,24,26,34,35,40,42,43,46,48]. Altogether, it seems reasonable to assume that the administration of SGLT2 inhibitors may have different effects in animal models of different diseases in different animal strains; and, in any case, the effect of these drugs could be influenced by the duration of the disease and by the duration of the treatment.

As described in Table 1, Table 2, Table 3 and Table 4, the nephroprotective effect of SGLT2 inhibitors, in the absence of diabetes, can be clearly appreciated in a number of renal diseases that are apparently independent of each other. The finding is surprising and could be easily explained only if these diseases would share, as a pathogenic mechanism, an increased SGLT2 activity. This assumption seems to be improbable, in particular when we take into account that glycosuria has never been associated with the diagnosis of most of the above-described renal diseases.

Interestingly, in the last few years, SGLT2 inhibitors have been shown to protect from inflammation, fibrosis, oxidative stress and sympathetic nervous over-activity. All of these dysfunctions are systemic and substantially independent from the activation or inhibition of SGLT2. These findings strongly support the alternative hypothesis that SGLT2 inhibitors might have one or more independent, and still unknown, activities not restricted to the kidney, and this could explain not only their “hyperglycemia-independent” renal effects but also their extra-renal effects that have been recently demonstrated.

The identification of the “alternative” mechanism(s) of action specific to SGLT2 inhibitors that end(s) up by protecting and preserving the renal function in the different (and, apparently, totally unrelated) kidney diseases described above could have a major impact from a clinical point of view. 

A potential key point concerns the side effects of this class of drugs. The ones related to their “canonical” glucose-lowering effect are well known and, as described above, relatively moderate. Side effects related to the alternative ways of action of SGLT2 inhibitors are, instead, yet poorly understood, and this could represent a problem to be solved, in particular in the case of a future clinical translation of these “alternative ways of action”.

In conclusion, only by solving this aspect will it be possible to clarify in full the potentiality of these drugs and to translate and implement their glucose-independent properties into clinical practice.

## Figures and Tables

**Table 1:** Experimental models using SGLT2 inhibitor empagliflozin to treat kidney disease.

Experimental Models of Kidney Disease	SGLT2 Inhibitor	Renal Functional Parameters	Kidney Tissue	References
Angiotensin II-dependent hypertension (Sprague Dawley rats)	Empagliflozin	↔ Glomerular filtration rate	↓ fibrosis ↓ inflammatory cells ↓ type I, type IV collagen	[20]
Angiotensin II-dependent hypertension (Wistar rats)	Empagliflozin +AT1 antagonist	↑ Glomerular filtration rate ↓ proteinuria	↑ SGLT2, ↓ ROS	[21]
Hypertensive rats (fawn-hooded rats)	Empagliflozin	↔ plasma creatinine ↔ plasma MCP-1 ↔ proteinuria	↑ kidney weight ↔ oxidative stress	[22]
2kidney-1clip hypertensive rats (Wistar rats)	Empagliflozin	↔ plasma creatinine ↔ plasma MCP-1 ↑ proteinuria	↔ kidney weight ↔ oxidative stress	[22]
Salt-sensitive hypertension in uninephrectomized (Hannover Sprague Dawley rats)	Empagliflozin	↑ plasma MCP-1 ↔ proteinuria	↑ kidney weight ↔ oxidative stress	[22]
Salt-sensitive hypertension in uninephrectomized (Sprague Dawley rats)	Empagliflozin	↑ Glomerular filtration rate ↑ Proteinuria	↑ HIF-1 ↓ inflammatory cells	[23]
Uninephrectomy-Doca-salt (different mouse strain)	Empagliflozin	↔ Glomerular filtration rate ↔ albuminuria	↔ fibrosis	[24]
Insulin resistance (Wistar rats)	Empagliflozin	↔ serum creatinine ↔ serum BUN	↓ fibrosis	[25]
Oxalate-induced nephropathy (C57BL/6N mice)	Empagliflozin	↔ plasma creatinine ↔ plasma BUN ↔ Glomerular filtration rate	↔ interstitial fibrosis	[26]
Cyclosporine nephropathy (Sprague Dawley rats)	Empagliflozin	↑ Glomerular filtration rate	↓ fibrosis ↓ inflammatory cells ↓ type I collagen ↓ type IV collagen	[27]
Unilateral ureteric obstruction (Wistar rats)	Empagliflozin	↓ serum BUN ↓ serum creatinine	↓ interstitial fibrosis ↓ inflammation ↓ tubular damage ↑ kloto expression	[28]
Subtotally nephrectomized rats (Wistar rats)	Empagliflozin	↔ Glomerular filtration rate ↔ albuminuria	↓ interstitial fibrosis ↓ glomerulosclerosis	[29]
Subtotally nephrectomized rats (Wistar rats)	Empagliflozin	↓ serum creatinine ↓ serum BUN ↓ albuminuria	↓ glomerulosclerosis ↓ interstitial fibrosis ↓ M2 macrophage polarization	[30]
Cardiorenal syndrome (Sprague Dawley rats)	Empagliflozin	↓ plasma creatinine ↓ proteinuria	↓ tubular damage ↓ Bowman’s capsule dilatation	[31]

↑ = increase, ↓ = decrease, ↔ = no significant change.

**Table 2:** Experimental models using SGLT2 inhibitor dapagliflozin to treat kidney disease.

Experimental Models of Kidney Disease	SGLT2 Inhibitor	Renal Functional Parameters	Kidney Tissue	References
Salt-sensitive hypertension (female Dahl SS rats)	Dapagliflozin	↔ Glomerular filtration rate	↓ fibrosis	[32]
Salt-sensitive hypertension (Dahl SS rats)	Dapagliflozin	↓ albuminuria	↓ fibrosis ↓ inflammation ↓ oxidative stress ↑ protective RAS signaling	[33]
Polycystic kidney disease (PKC rats)	Dapagliflozin	↑ Glomerular filtration rate ↑ albuminuria	↑ cyst volume	[34]
Polycystic kidney disease (Han:SPRD rats)	Dapagliflozin	↑ Glomerular filtration rate ↓ albuminuria	↔ cyst volume ↔ fibrosis ↔ inflammatory cells	[35]
Prediabetic rats (Wistar rats)	Dapagliflozin	↓ Glomerular hyperfiltration ↓ albuminuria	↓ fibrosis ↓ inflammation ↓ endoplasmatic reticulum stress ↓ apoptosis	[36]
High-fat/high-fructose-diet-induced insulin resistance (Wistar rats)	Dapagliflozin	↓ serum creatinine	↓ fibrosis ↓ inflammation ↓ oxidative stress ↓ apoptosis ↓ triglyceride accumulation	[37]
Gentamicin-induced nephrotoxicity (Wistar rats)	Dapagliflozin	↓ serum creatinine ↓ serum BUN ↓ proteinuria	↓ tubular and glomerular injury ↓ oxidative stress ↓ apoptosis	[38]
Protein overload- proteinuria (C57BL/6N mice)	Dapagliflozin	↔ Glomerular filtration rate ↓ proteinuria	↓ glomerular lesions ↓ inflammation	[39]
Adriamicin-induced nephropathy (balb/c mice)	Dapagliflozin	↔ proteinuria	↔ fibrosis ↔ inflammation ↔ tubular atrophy	[40]
Renal ischemia- reperfusion injury (C57BL/6 mice)	Dapagliflozin	↓ serum creatinine ↓ serum BUN	↓ tubulointerstitial lesions ↓ apoptosis ↑ HIF-1 expression	[41]
Subtotally nephrectomized rats (Sprague Dawley rats)	Dapagliflozin	↔ Glomerular filtration rate ↔ proteinuria	↔ glomerulosclerosis ↔ interstitial fibrosis ↔ inflammatory cells	[42]
Subtotally nephrectomized rats (Sprague Dawley rats)	Dapagliflozin	↔ Glomerular filtration rate ↔ proteinuria	↔ glomerulosclerosis ↔ inflammatory cells	[43]

↑ = increase, ↓ = decrease, ↔ = no significant change.

**Table 3:** Experimental models using SGLT2 inhibitors (phlorizin, ipragliflozin, luseogliflozin, TA-1887) to treat kidney disease.

Experimental Models of Kidney Disease	SGLT2 Inhibitor	Renal Functional Parameters	Kidney Tissue	References
Polycystic kidney disease (Han:SPRD rats)	Phlorizin	↑ Glomerular filtration rate ↓ albuminuria	↓ cyst volume	[44]
Adenine-induced chronic kidney disease (C57BL/6JJcl mice)	Ipragliflozin	↓ plasma creatinine	↓ fibrosis ↓ tubular dilatation ↓ oxidative stress	[45]
Adenine-induced chronic kidney disease (Wistar rats)	Luseogliflozin	↔ serum BUN	↔ tubular dilation	[46]
Renal ischemia- reperfusion injury (C57BL/6J mice)	Luseogliflozin	↑ Glomerular filtration rate	↓ interstitial fibrosis ↓ capillary rarefaction	[47]
Subtotally nephrectomized rats (Sprague Dawley rats)	TA-1887	↔ plasma creatinine ↔ plasma BUN ↔ Glomerular filtration rate ↔ albuminuria	↔ interstitial fibrosis ↔ glomerular injury ↔ RAS	[48]

↑ = increase, ↓ = decrease, ↔ = no significant change.

**Table 4:** Experimental models using SGLT2 inhibitor canagliflozin to treat kidney disease.

Experimental Models of Kidney Disease	SGLT2 Inhibitor	Renal Functional Parameters	Kidney Tissue	References
Cisplatin-induced nephrotoxicity (CD1 mice)	Canagliflozin	↑ Glomerular filtration rate ↓ albuminuria	↓ tubular and glomerular injury ↓ oxidative stress	[49]
Cisplatin-induced nephrotoxicity (C57BL/6 mice)	Canagliflozin	↓ serum creatinine ↓ serum BUN	↓ tubular injury ↓ apoptosis	[50]
Adenine-induced chronic kidney disease (Wistar rats)	Canagliflozin	↓ plasma creatinine ↓ plasma BUN ↓ albuminuria	↓ fibrosis ↓ inflammation ↓ oxidative stress	[51]
Isoprenaline-induced renal oxidative damage (Long Evans rats)	Canagliflozin	↓ plasma creatinine	↓ fibrosis ↓ inflammatory cells ↓ oxidative stress ↓ apoptosis	[52]

↑ = increase, ↓ = decrease, ↔ = no significant change.

## Data Availability

Data sharing is not applicable.
